# Height, adiposity and hormonal cardiovascular risk markers in childhood: how to partition the associations?

**DOI:** 10.1038/ijo.2014.24

**Published:** 2014-03-04

**Authors:** J C K Wells, T J Cole

**Affiliations:** 1 Childhood Nutrition Research Centre, UCL Institute of Child Health, London, UK; 2 Centre for Paediatric Epidemiology and Biostatistics, UCL Institute of Child Health, University College, London, UK

**Keywords:** BMI, cardiovascular risk, size adjustment, adiposity, leptin, insulin resistance

## Abstract

**Objective:**

Obesity is associated with rapid growth during childhood. There is uncertainty over how to adjust for body size, when using adiposity as a proxy for cardiovascular risk. We studied associations of height, body composition (by dual-energy X-ray absorptiometry) and cardiovascular risk markers (insulin resistance (IR), leptin) in children.

**Methods:**

Using partial correlations in 172 children aged 7–12 years, we investigated associations of (a) fat mass with IR or leptin, adjusting for height or lean mass, and (b) height or lean mass with IR or leptin, adjusting for fat mass. Analyses were conducted both cross-sectionally at each age, and for changes between 7 and 12 years.

**Results:**

Height, fat mass, lean mass, IR and leptin were all inter-correlated at all ages. Although fat mass was strongly associated with IR and leptin, height was independently negatively associated with leptin (whole sample, adjusting for age: boys *r*=−0.12, girls *r*=−0.13; *P*<0.001). Independent of adiposity, height was also associated with insulin IR (whole sample, adjusting for age: boys *r*=0.11, girls *r*=0.20; *P*<0.001). When analysed by year of age, these associations tended to remain significant at older ages. Change in height from 7 to 12 years was also associated with change in IR (boys: *r*=0.18, *P*<0.05; girls: *r*=0.34, *P*<0.01), independently of change in adiposity, with similar findings for lean mass.

**Conclusions:**

During childhood, markers of cardiovascular risk have a complex profile, associated with growth as well as fat accumulation. Taller and faster-growing children have elevated risk markers, independently of their adiposity. These findings have implications for the interpretation of pediatric indices of adiposity that aim to adjust for body size. Adiposity indices that perform best at summarizing metabolic risk may not be those that perform best at understanding the developmental aetiology of risk.

## Introduction

Obesity is now well established to elevate the risk of chronic degenerative diseases, including type 2 diabetes, hypertension, cardiovascular disease (CVD) and some cancers.^1,2^ Although the main effects of these diseases are experienced in later life, childhood obesity appears to increase both short- and long-term health risks.^3, 4, 5^ Monitoring of nutritional status during childhood may potentially contribute to the reduction of these risks.

To address this issue, epidemiological studies have researched both the factors that predispose to childhood obesity, and the profile of CVD risk accumulation over time. In this context, the most widely studied index of nutritional status has been body mass index (BMI).^6, 7, 8^ However, BMI does not provide information on the relative proportions of fat and lean components in weight,^9^ hence, there is also interest in studying the accretion of fat and lean tissue through childhood, and exploring their association with the development of risk.^10, 11, 12^

The primary body composition outcomes investigated when ‘going beyond' BMI are fat mass and lean mass (used here synonymously with fat-free mass). However, it has long been recognized that such data require some form of correction for body size.^13, 14, 15^ Individuals who have grown faster or for longer are expected to have greater absolute tissue masses, hence, size adjustment is required to assess whether a given individual is fatter or just larger. Whereas BMI adjusts weight for height, body composition data have conventionally been expressed in the format of ‘percentage fat' (%fat), which describes the proportion of fat in weight.^13^

The use of %fat as an index of adiposity presents two problems, which remain poorly recognized. The first issue is that this approach directs undue attention to fat at the expense of lean. This is unfortunate, as while excess fat is associated with poorer health, variability in lean mass also contributes to the metabolic profile.^16, 17, 18^ The second issue is that %fat is statistically problematic, as fat mass, when divided by weight, is present in both numerator and denominator.^19,20^ As absolute fat mass increases, %fat rises increasingly slowly, eventually trending towards an asymptote at around 60% fat. In obese individuals, even large gains or losses in adipose tissue mass may induce only small changes in %fat. For example, if a person weighing 100 kg and comprising 50% fat gains 10 kg of fat, the 20% increase in absolute fat mass seems very modest when expressed as a change in %fat, from 50 to 54.5%. Furthermore, high %fat values may reflect high adiposity and/or low lean mass.^19^ The proportion of fat in weight is therefore not an index of adiposity that is independent of body size.

In adults, many researchers resolve this problem by splitting BMI into two components, the lean mass index (LMI: lean mass/height-squared) and the fat mass index (FMI: fat mass/height-squared), each expressed in the same kg m^−^^2^ units as BMI. This approach, developed by Van Itallie *et al.*,^14^ has two benefits: it adjusts tissue masses for an independent component of body size, while also keeping fat and lean outcomes separate. When this approach is adopted in children, however, an additional statistical issue becomes relevant.

BMI is calculated as weight/height-squared because this statistical adjustment has been found to generate an index of weight that is broadly independent of height. In some children's age groups, however, BMI may remain correlated with height.^21,22^ This could suggest that taller children are heavier even after standardizing for height, or it could indicate that statistical adjustment using the square of height is incompletely effective. Each of these arguments has been made.^19,23^ The issue becomes even more important if body composition is considered. Whereas lean mass tends to scale with height-squared in childhood as in adulthood,^19,20,24^ the power (*n*) to which height must be raised to generate an index fat mass/height^*n*^ that is independent of height approaches 5 or 6 in childhood,^19^ although it may also vary with age. Fat mass index therefore remains correlated with height, that is, taller children have greater adiposity using this approach.^19,23^

Recently, this dilemma received attention in analyses of the EarlyBird cohort of children in Plymouth, UK, where data have been collected at sequential time points on each of body size, body composition and metabolic risk.^25^ These data allow investigation of how body composition data might best be expressed, in order to index CVD risk. In a recent publication on this cohort, Metcalfe *et al.^25^* reported that BMI or FMI had stronger associations with leptin and insulin resistance (IR) than did indices of weight or fat mass that adjusted completely for height variability. They therefore proposed that height-normalized indices were not the optimal approach for expressing adiposity data. Instead, they argued that it is more appropriate to accept that taller children are fatter, and that it does not help to use statistical adjustments to hide this association.

In an invited commentary, we suggested that it may still be helpful to try to disentangle associations of size and adiposity with CVD risk, as each exposure may provide different information about the aetiological development of risk.^26^ If BMI indexes risk, but also remains positively correlated with height, then it appears that some of this metabolic risk derives from height and growth trajectory, independent of the risk deriving from adiposity.

As yet, however, the independent respective associations of fat and height with indicators of cardiometabolic risk have not been clearly determined. To enable investigation of this issue, Metcalfe *et al.*^25^ made their data available to us. In this paper, we therefore explore the independent associations of body size and body composition with CVD risk in the EarlyBird cohort. The aim was to gain greater insight into how these different exposures interact in accounting for CVD risk, and hence to improve understanding of what analyses may be done in future studies and how data may best be expressed.

## Materials and Methods

Data were available annually from 7 to 12 years for 152 boys and 120 girls, with a small loss of sample size (~15 boys, ~20 girls, varying by trait) over the study period (see Table 1). The traits examined here were weight and height, fat mass and lean mass, measured by dual-energy X-ray absorptiometry, and fasting blood concentrations of insulin and leptin, two markers of CVD risk. The insulin data were obtained through a validated approach, HOMA2-IR, and represent a marker of peripheral IR. The details of the study design and data collection have been described previously.^25^

### Analyses

Preliminary analyses showed that fat mass, leptin and IR were right-skewed, hence these variables were natural log-transformed to achieve normality. All subsequent analyses were conducted on these log-transformed values. All analyses were also conducted separately for boys and girls.

Crude Pearson's correlations were calculated to establish associations of height with body composition, leptin and IR and also of fat mass and lean mass with leptin and IR. These correlations were first calculated for each age group separately, and then for all data combined, partialling out the effect of age. We also tested whether the sexes showed different associations between height and leptin or insulin resistance, by including a sex-interaction term.

Partial correlations were then calculated to establish the association of fat with CVD risk adjusting for height, and of height with CVD risk adjusting for fat. A similar approach was then used for body composition to establish the association of fat mass with CVD risk adjusting for lean, and of lean mass with CVD risk adjusting for fat. As BMI remains the index of adiposity most commonly used in large epidemiological studies, we also calculated the associations of height and lean mass with CVD risk, partialling out the effect of BMI. Again, these correlations were first calculated for each separate age group and then for all age groups combined, partialling out the effect of age.

We then calculated the change over time in each variable, as the difference between the 7-year and 12-year values. These changes are termed Δ, for example Δ fat refers to the increase in fat mass between 7 and 12 years. These changes were calculated from the log-transformed start and end values for fat mass, leptin and IR. We first tested whether baseline leptin and IR were correlated with Δ height, Δ lean or Δ fat. We then calculated partial correlations to assess associations of Δ fat with CVD risk adjusting for Δ height or Δ lean, or of Δ height or Δ lean with Δ CVD risk adjusting for Δ fat.

## Results

**Figure 1:**
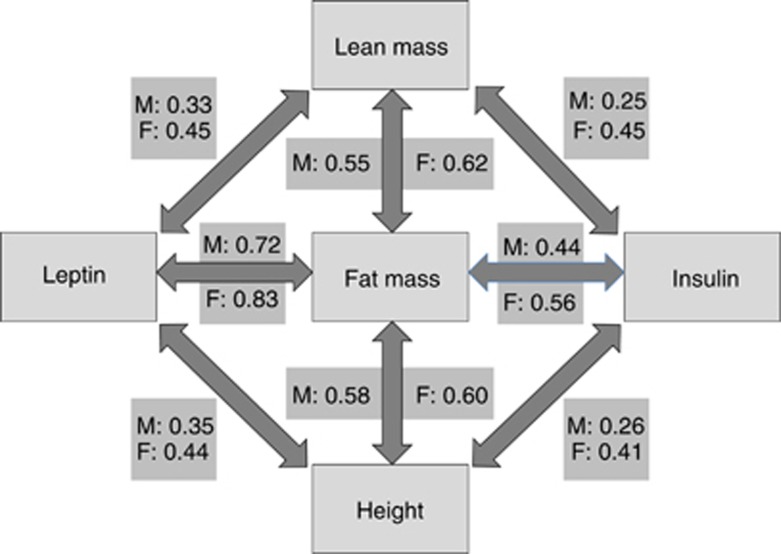
Age-adjusted correlations of height, lean mass or fat mass with leptin and insulin in the entire sample, presented separately for males (M) and females (F). Fat mass, insulin and leptin were natural log-transformed. All correlations *P*<0.0001.

Descriptive data on the cohort have been given previously.^25^ Height was significantly positively correlated with weight, BMI, fat and lean masses, leptin and IR at all ages in both sexes (Table 1). Thus, at any age, taller children have greater BMI and absolute tissue masses, and greater circulating levels of leptin and IR. In the whole sample, there were sex interactions in the association of leptin and IR with height, indicating that the correlation coefficients were significantly greater in girls than in boys (leptin: *P*<0.001; IR: *P*<0.02). Supplementary Table S1 further shows that fat mass and lean mass were both positively associated with leptin and IR at all ages, and significantly so at most ages. Figure 1 summarizes the age-adjusted correlations of the three components of body size and composition (height, lean mass and fat mass) with the two metabolic outcomes (leptin and IR). All associations were highly significant in both sexes, *P*<0.0001.

Table 2 shows that at all ages, fat mass adjusted for height was correlated with both leptin and IR. In addition, height adjusted for fat mass was inversely associated with leptin. In boys, these correlations were significant at 7, 11 and 12 years and in the age-adjusted whole sample, while in girls, the correlations were significant at 8 and 10 years and in the age-adjusted whole sample. In girls but not boys, height adjusted for fat mass was positively associated with IR at 10 and 12 years. Table 2 also shows the associations of height with IR and leptin, partialling out the index of adiposity most widely used in clinical practice and research studies, BMI. Height shows independent associations with IR in both sexes, with the associations stronger at older ages. There is only weak evidence of an association of height with IR after adjustment for fat, and this association is positive, whereas that of height and leptin adjusted for fat mass is negative.

Table 3 shows that at all ages, fat mass adjusted for lean mass was correlated with leptin and IR. In boys at 11 and 12 years, and in girls at 8, 10 and 11 years, lean mass adjusted for fat mass was inversely associated with leptin. In girls aged 11 and 12 years, lean mass adjusted for fat mass was positively associated with IR.

Table 4a shows that baseline leptin and IR were not associated with Δ height in either sex. Baseline leptin was directly associated with Δ lean in girls, and inversely associated with Δ fat in both sexes. In girls, baseline IR was directly associated with Δ lean, and inversely associated with Δ fat. Table 4b also shows correlations for changes (Δ) in the variables from 7 to 12 years. Δ BMI was positively associated with changes in all other traits. Δ height was positively associated with Δ lean and Δ fat, showing that children growing faster in height gained more fat and more lean. However, Δ lean and Δ fat were not correlated. Both Δ height and Δ lean were associated with Δ IR but not Δ leptin, whereas Δ fat was associated with both Δ leptin and Δ IR. Furthermore, Δ leptin was positively associated with Δ IR.

Table 5 shows partial correlations, adjusting the associations between Δ fat and Δ cardiovascular risk markers for Δ height or Δ lean mass, and vice versa. Adjusting for Δ height, Δ fat was positively associated with Δ IR and Δ leptin. Similarly, adjusting for Δ lean, Δ fat was positively associated with both Δ leptin and Δ IR. Thus, independently of growth in height or lean mass, increasing adiposity was associated with increased CVD risk. Adjusting for Δ fat, Δ height was associated with Δ IR but not with Δ leptin, while Δ lean was associated with Δ IR in both sexes and showed a borderline-significant association with Δ leptin in girls only. These results indicate that independent of increases in adiposity, growth in height or lean mass was associated with increasing IR.

## Discussion

These data indicate that taller children have greater absolute fat mass and leptin and IR, as described previously in the same cohort, with our analyses demonstrating that the crude association of height with leptin and IR is stronger in girls than in boys.^25^ However, height and lean mass also show associations with each of fat mass, leptin and IR. Disentangling their respective independent associations may help understand how best to express data on body composition in order to index and interpret metabolic risk.

Metcalf *et al.*^25^ argued that normalizing weight or fat mass for height ‘obfuscates', and that the elevated metabolic risk of taller fatter children would be missed using this approach. We argued that part of the elevated risk in taller children might derive from their height, independently of their high adiposity, and that these different sources of risk are important to know about.^26^

Consistent with our predictions, our cross-sectional analyses show that while much of the variability in CVD risk can be attributed to adiposity, height also has associations with each of leptin (both sexes) and IR (girls only), that are independent of adiposity. From 11 years in boys, and at 8 and 11 years in girls, taller children have lower leptin, and taller girls at 10 and 12 years have greater IR. These trends are also significant in the whole sample, adjusting for age. These findings are broadly replicated for lean mass, such that adjusting for adiposity, older children with greater levels of lean mass have lower leptin, whereas older girls with more lean mass have greater IR.

The strong association of fat mass with leptin at every age, even after adjusting for height, is consistent with the notion that leptin acts as a signal of adiposity.^27,28^ The negative association of leptin with lean mass or height, which only emerged after adjusting for adiposity, suggests that leptin might further differentiate *between* fat and lean tissues, such that for a given quantity of fat mass, those with greater lean mass produce less leptin. Our longitudinal analyses show that baseline leptin is associated with the subsequent increase in lean mass, significant in girls only. However, baseline leptin did not predict increases in height, and the main longitudinal associations were a positive correlation between change in fat and change in leptin, indicating that children increasing in adiposity secrete more leptin. Thus, leptin may reflect the ratio of fat to lean as well as energy stores *per se*, without driving subsequent tissue accretion patterns over time. One possibility is that leptin acts as a permissive signal for puberty,^29^ such that children progressing faster through puberty are both taller and also have lower leptin, as the energy is already being invested in the pubertal growth spurt.

In longitudinal analyses, change in adiposity was strongly associated with greater change in IR, but the causal direction of this association remains unclear. It may derive either through IR driving the accumulation of adipose tissue,^30,31^ or through weight gain and a lipogenic diet favouring IR to protect tissues against nutrient overload.^32,33^ Regardless of the causal direction of the association, it is clear that increases in adiposity are the dominant factor correlating with IR in this sample, however, gains in height and lean tissue also showed independent associations with increases in IR.

Although IR was associated in cross-sectional analyses with height and lean mass at every age in crude analyses, these associations disappeared once adjusted for adiposity, except in older girls. Thus, we assume that younger taller children have greater IR because they are also fatter, whereas older taller girls have greater IR independent of their fatness. Higher IR at 7 years predicted greater gains in lean mass in girls, but not greater increase in height, whereas the increase in IR between 7 and 12 years was correlated with increases in each of height, lean mass and fat mass. Although it remains difficult to interpret these data in terms of causality, our analyses demonstrate that growing taller and depositing more lean tissue are associated with increasing IR independent of adiposity, hence, fasting insulin by HOMA acts as a marker of growth as well as adiposity.

A limitation of our study is that we were only able to analyse two markers of cardiovascular risk, namely leptin and IR. We focused on these two outcomes precisely because they are simultaneously regulators of growth and body composition, while also predictive of future chronic disease risk. We cannot be sure that other cardiovascular risk markers would show similar associations. However, in a previous analysis of the ALSPAC cohort at age 9 years, we showed independent associations of height, lean mass and adiposity with blood pressure.^18^ Further work will be required to reveal how general the association of height and cardiovascular risk is in children. We also note that in adults, short stature is associated with greater cardiovascular risk.^34,35^ This contrast may arise as taller children may be those growing faster during childhood, and not necessarily those who will be tallest in adulthood.

A second limitation was the sample size and the conducting of many statistical tests, which increases the possibility of reporting significant associations that actually arose through chance. Our findings should therefore be confirmed using larger data sets, however, we note that our previous study, identifying independent associations of height and adiposity with blood pressure, was conducted in >6500 children.^18^

Overall, our analyses indicate complexity in the associations between body size, growth rate, body composition and metabolic risk. Further work will need to be undertaken to tease out the effects in greater detail, but it is already clear from these data that associations of height or lean mass with CVD risk are not fully explained by their respective associations with adiposity. Rather, tall height and high lean mass are associated with CVD risk, independent of adiposity, and these associations evolve over time.

As both height and adiposity correlate with elevated metabolic risk, some indices such as BMI or FMI incorporate both of these sources of risk without distinguishing between them.^26^ Table 2 shows that height retains associations with IR, after adjusting for BMI, but this information is lost when using BMI as an index of adiposity, as the rationale of the index is to ‘remove any effect of height'. The same is true for FMI, which is generally treated only as an index of adiposity.

Previously, we noted that fat mass divided by height^6^ was independent of height in children aged 8 years.^19^ Using such an index, it is possible to assess the proportion of risk that derives from adiposity, independent of that deriving from height. This partitioning of risk has some benefits, because the factors (environmental or genetic) that account for variability in adiposity may not be identical with those that account for variability in growth.^26^ For example, linear growth appears most sensitive to environmental factors during fetal life and infancy,^36,37^ and many studies have associated early catch-up growth with subsequent adiposity.^12^^38, 39, 40^ However, adiposity may be more sensitive to behaviours such as diet composition and physical activity behaviour throughout childhood.^41, 42, 43, 44^

In general, the management of obesity favours loss of fat, but not a reduction in growth,^45^ hence, it is possible that the treatment of taller children might be inherently less successful at reducing metabolic risk than the treatment of shorter children. In Swedish adults, for example, the metabolic penalty (elevated blood pressure) for low birth weight was greater in individuals with both high BMI and greater height, and the highest blood pressures were found in those born small who were both obese and tall in adulthood.^46^

However, the partitioning of CVD risk into different components is also artificial, because of the complex ways in which growth in height and adiposity appear to be connected, in part by the very hormones that are used as markers of metabolic risk. As the hormones that index CVD risk also contribute to the regulation or growth and tissue deposition, BMI and FMI may be clinically useful ways to summarize metabolic risk, but not necessarily the best way to understand its aetiology.

Nevertheless, we suggest that such partitioning may be particularly helpful when addressing children approaching and undergoing puberty. At this time, elevations of CVD risk may relate in part to the faster tempo of growth during this period, and it may be valuable to quantify the component of risk attributable to adiposity, potentially more amenable to management. A novel statistical approach (SITAR)^47^ that can disentangle size, growth tempo and velocity might clarify these associations, for example, by adjusting the growth trajectory for the timing of puberty. Each approach therefore has some merits: BMI or FMI give a global index of CVD risk, integrating effects of growth and adiposity, whereas approaches using multiple regression analysis (simply entering terms separately) or specific indices (for example, fat normalized for height, or regression residuals) may tease out the contribution of growth versus adiposity to CVD risk, and clarify the opportunities for intervention.

## Figures and Tables

**Table 1:** Crude correlations of height with indices of weight, body composition and metabolic risk

	*N*	*Weight*	*BMI*	*Fat mass*	*Lean mass*	*Leptin*	*Insulin resistance*
*Boys*							
Visit							
7	152	0.81	0.34	0.59	0.87	0.32	0.19*
8	149	0.80	0.41	0.55	0.82	0.38	0.21*
9	138	0.81	0.47	0.61	0.83	0.34	0.36
10	140	0.80	0.50	0.62	0.81	0.43	0.41
11	134	0.77	0.46	0.60	0.81	0.31	0.21*
12	135	0.75	0.43	0.49	0.81	0.32	0.31
All adj. age	751	0.73	0.42	0.58	0.84	0.35	0.26

*Girls*							
Visit							
7	120	0.80	0.42	0.62	0.88	0.47	0.36
8	102	0.80	0.49	0.65	0.89	0.44	0.44
9	110	0.79	0.49	0.63	0.88	0.42	0.37
10	110	0.78	0.51	0.62	0.87	0.48	0.51
11	95	0.74	0.47	0.55	0.82	0.43	0.46
12	97	0.75	0.49	0.62	0.84	0.53	0.49
All adj. age	574	0.75	0.49	0.60	0.84	0.44	0.41

Abbreviation: BMI, body mass index.

Fat mass, leptin and insulin resistance are natural log-transformed.

All adj. age—correlation in whole sample, partialling out the effect of age.

All correlations are significant *P*<0.001 except *P<0.025.

**Table 2:** Partial correlations for associations of leptin and insulin with fat adjusted for height, and with height adjusted for fat or BMI

	*Leptin*			*Insulin*		
	*Fat adj. height*	*Height adj. fat*	*Height adj. BMI*	*Fat adj. height*	*Height adj. fat*	*Height adj. BMI*
*Boys*						
Visit						
7	0.68**	−0.20**	0.05	0.28**	−0.02	0.05
8	0.61**	−0.07	0.12	0.41**	−0.10	0.07
9	0.53**	−0.02	0.09	0.32**	0.12	0.21*
10	0.66**	−0.04	0.12	0.41**	0.13	0.23*
11	0.82**	−0.36**	−0.04	0.40**	−0.07	0.04
12	0.84**	−0.29**	0.03	0.41**	0.04	0.16
All adj. age	0.68**	−0.12**	0.09*	0.37**	0.01	0.11**

*Girls*						
Visit						
7	0.70**	−0.02	0.21*	0.47**	−0.00	0.14
8	0.79**	−0.19 **	0.10	0.38**	0.15	0.25*
9	0.74**	−0.27	0.08	0.38**	0.02	0.15
10	0.76**	−0.06	0.20*	0.43**	0.20*	0.32**
11	0.88**	−0.34**	0.04	0.55**	0.13	0.22*
12	0.88**	−0.07	0.19	0.41**	0.22 *	0.28**
All adj. age	0.78**	−0.13**	0.12*	0.52**	0.11*	0.20**

Abbreviation: BMI, body mass index.

Fat mass, leptin and insulin are natural log-transformed.

All adj. age—correlation in whole sample, partialling out the effect of age.

**P<0.001; *P<0.05.

**Table 3:** Partial correlations for associations of leptin and insulin with fat adjusted for lean, and with lean adjusted for fat

	*Leptin*		*Insulin*	
	*Fat adj. lean*	*Lean adj. fat*	*Fat adj. lean*	*Lean adj. fat*
*Boys*				
Visit				
7	0.63**	−0.10	0.18 *	0.13
8	0.60**	−0.01	0.42**	−0.11
9	0.57**	−0.11	0.36**	0.05
10	0.67**	−0.04	0.43**	0.08
11	0.81**	−0.28*	0.42**	−0.10
12	0.86**	−0.41**	0.41**	0.05
All adj. age	0.68**	−0.13**	0.38**	−0.01

*Girls*				
Visit				
7	0.69**	−0.04	0.44**	0.02
8	0.80**	−0.23*	0.36**	0.20
9	0.70**	−0.12	0.34*	0.03
10	0.77**	−0.20*	0.40**	0.16
11	0.87**	−0.32*	0.48**	0.25*
12	0.86**	−0.03	0.34*	0.29**
All adj. age	0.78**	−0.13**	0.40**	0.17**

Fat mass, leptin and insulin are natural log-transformed.

All adj. age—correlation in whole sample, partialling out the effect of age.

**P<0.0001; *P<0.05.

**Table 4a:** Longitudinal associations between body composition and metabolic risk from 7 to 12 years. Correlations between baseline leptin or insulin and change in size or body composition between 7 and 12 years

	*Δ Height*		*Δ Lean*		*Δ Fat*	
	*Boys*	*Girls*	*Boys*	*Girls*	*Boys*	*Girls*
Leptin 7 years	0.06	0.15	0.15	0.40**	−0.21**	−0.27**
Insulin 7 years	−0.03	0.10	0.02	0.27*	0.06	−0.35**

Fat mass, leptin and insulin are natural log-transformed.

Δ=change between 7 and 12 years.

**P<0.01; *P<0.05.

**Table 4b:** Longitudinal associations between body composition and metabolic risk from 7 to 12 years. Correlations of change in size, body composition and metabolic risk from 7 to 12 years

	*Δ BMI*		*Δ Lean*		*Δ Fat*		*Δ Leptin*		*Δ Insulin resistance*	
	*Boys*	*Girls*	*Boys*	*Girls*	*Boys*	*Girls*	*Boys*	*Girls*	*Boys*	*Girls*
Δ Height	0.37^#^	0.46^#^	0.77^#^	0.80^#^	0.27^#^	0.20*	0.12	0.17	0.23**	0.36^#^
Δ BMI	—	—	0.46^#^	0.58^#^	0.81^#^	0.70^#^	0.67^#^	0.57^#^	0.38^#^	0.47^#^
Δ Lean	—	—	—	—	0.15	0.08	0.04	0.18	0.29^#^	0.39^#^
Δ Fat	—	—	—	—	—	—	0.68^#^	0.56^#^	0.25**	0.41^#^
Δ Leptin	—	—	—	—	—	—	—	—	0.44^#^	0.50^#^

Abbreviation: BMI, body mass index.

Fat mass, insulin and leptin are natural log-transformed.

^#^*P*<0.001; **P<0.01; *P<0.05.

**Table 5:** Partial correlations of change in size, body composition and metabolic risk from 7 to 12 years

	Δ Leptin adj. Δ Height		Δ Insulin adj. Δ Height	
	Boys	Girls	Boys	Girls
Δ Fat	0.68^#^	0.55^#^	0.20*	0.36^#^
	Δ Leptin adj. Δ Fat		Δ Insulin adj. Δ Fat	
	Boys	Girls	Boys	Girls
Δ Height	−0.09	0.09	0.20*	0.31**
	Δ Leptin adj. Δ Lean		Δ Insulin adj. Δ Lean	
	Boys	Girls	Boys	Girls
Δ Fat	0.68^#^	0.57^#^	0.22*	0.41^#^
	Δ Leptin adj. Δ Fat		Δ Insulin adj. Δ Fat	
	Boys	Girls	Boys	Girls
Δ Lean	−0.08	0.19	0.21*	0.39^#^

Fat mass, insulin and leptin are natural log-transformed.

Δ=change between 7 and 12 years.

^#^*P*<0.001; **P<0.01; *P<0.05.
