# Supplementary material for: Height, adiposity and hormonal cardiovascular risk markers in childhood: how to partition the associations?
Source: Int J Obes (Lond). 2014 Mar 4;38(7):930–5. doi: 10.1038/ijo.2014.24 (PMC4088335; doi:10.1038/ijo.2014.24)
Supplement: Supplementary online Table 1 [file ijo201424x1.doc]

**Supplementary Table S1. Crude correlations of body composition with metabolic risk**

Fat mass Lean mass

Leptin Insulin Leptin Insulin

Boys

Visit

7 0.70 0.30 0.40 0.28

8 0.68 0.42 0.35 0.17

9 0.61 0.46 0.29 0.31

10 0.74 0.54 0.43 0.39

11 0.82 0.45 0.38 0.20

12 0.84 0.49 0.23 0.27

All adj. age 0.72 0.44 0.33 0.25

Girls

Visit

7 0.77 0.57 0.45 0.40

8 0.83 0.55 0.43 0.47

9 0.78 0.55 0.48 0.43

10 0.82 0.59 0.49 0.50

11 0.88 0.65 0.40 0.55

12 0.92 0.59 0.64 0.57

All adj. age 0.83 0.56 0.45 0.45

Fat mass, leptin and insulin are natural log-transformed.

All adj. age – correlation in whole sample, partialling out effect of age

Age-specific analyses: correlations of 0.25 or greater p<0.001, and 0.20 or greater p<0.05
